# Haploinsufficiency of the *FOXA2* associated with a complex clinical phenotype

**DOI:** 10.1002/mgg3.1086

**Published:** 2020-04-11

**Authors:** Idris Mohammed, Sara Al‐Khawaga, David Bohanna, Abdusamea Shabani, Faiyaz Khan, Donald R. Love, Zafar Nawaz, Khalid Hussain

**Affiliations:** ^1^ College of Health & Life Sciences Hamad Bin Khalifa University Doha Qatar; ^2^ Division of Endocrinology Department of Pediatric Medicine Sidra Medicine Doha Qatar; ^3^ Department of Radiology Sidra Medicine Doha Qatar; ^4^ Division of Pathology Genetics Department of Pathology Sidra Medicine Doha Qatar; ^5^ Diagnostic Genomic Division Department of Laboratory Medicine and Pathology Hamad Medical Corporation Doha Qatar

**Keywords:** 20p11.2 deletion, *FOXA2*, growth hormone deficiency, haploinsufficiency, hypothyroidism

## Abstract

**Background:**

There are few reports describing the proximal deletions of the short arm of chromosome 20, making it difficult to predict the likely consequences of these deletions. Most previously reported cases have described the association of 20p11.2 deletions with Alagille syndrome, while there are others that include phenotypes such as panhypopituitarism, craniofacial dysmorphism, polysplenia, autism, and Hirschsprung disease.

**Methods:**

Molecular karyotyping, cytogenetics, and DNA sequencing were undertaken in a child to study the genetic basis of a complex phenotype consisting of craniofacial dysmorphism, ocular abnormalities, ectopic inguinal testes, polysplenia, growth hormone deficiency, central hypothyroidism, and gastrointestinal system anomalies.

**Results:**

We report the smallest described de novo proximal 20p11.2 deletion, which deletes only the *FOXA2* leading to the above complex phenotype.

**Conclusions:**

Haploinsufficiency of the *FOXA2* only gene is associated with a multisystem disorder.

## Graphical abstract

Haploinsufficiency of the *FOXA2* leads to a complex phenotype consisting of craniofacial dysmorphism, ocular abnormalities, ectopic inguinal testes, polysplenia, growth hormone deficiency, central hypothyroidism, and gastrointestinal system anomalies.

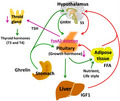

## 1 INTRODUCTION

The forkhead box A (FOXA) transcription factor plays an important role in multiple stages of life, from early development to endoderm formation, regulation of genes involved in growth and proliferation, fertility, organogenesis and differentiation, metabolism, homeostasis, and the immune system (Friedman & Kaestner, 2006; Kaestner, 2010; Kelleher et al., 2017). *FOXA2* expression occurs in the primitive streak and in the node of the embryo, which are both crucial for gastrulation. *FOXA2* expression is also active in the anterior axial mesoderm, definitive endoderm formation, as well as ectoderm‐derived neural tissues and endoderm‐derived tissues (pancreas, liver, thyroid, prostate, and lung) in early development and adulthood (Besnard, Wert, Hull, & Whitsett, 2004; Friedman & Kaestner, 2006; Kaestner, 2000).

β‐cell‐specific *FOXA2*‐knockout mice exhibit severe hyperinsulinemic hypoglycemia and hypoglucagonemia phenotype due to an increased insulin to glucagon ratio (3–4 fold), and die shortly after birth due to inhibition of notochord and endoderm formation (Gao et al., 2010; Lantz et al., 2004; Sund et al., 2001). The phenotypic outcome is likely due to the role that *FOXA2* plays in regulating the expression of genes in pancreatic β‐cells that are important in glucose sensing and insulin secretion, including *KCNJ11*, *ABCC8*, *GLUT2*, and *GCK* (Heddad Masson et al., 2014; Wang, Gauthier, Hagenfeldt‐Johansson, Iezzi, & Wollheim, 2002).

There are very few reports describing the constitutional heterozygous proximal deletions of chromosome 20 short arm (20p), making it difficult to predict phenotypic outcomes. Two reports have described inherited 20p deletions from normal mosaic carrier mothers: the first manifesting panhypopituitarism, and craniofacial dysmorphism (Garcia‐Heras, Kilani, Martin, & Lamp, 2005); and the second, a patient manifesting inadequate bile ducts, heart defect, corneal abnormality, and mild developmental delay (Laufer‐Cahana et al., 2002). Earlier studies have described patients with heterozygous 20p deletions with varying phenotypes including craniofacial dysmorphism, Hirschsprung disease, cognitive delay, autistic behavior, gastrointestinal anomalies, hypoglycemia, seizures, panhypopituitarism, endoderm‐derived organ abnormalities, and hearing loss (Dayem‐Quere et al., 2013; Garcia‐Heras et al., 2005; Kale, Patil, & Pandit, 2017; Kamath et al., 2009; Michaelis et al., 1997). Most reported chromosome 20p deletion cases constitute interstitial deletions involving the 20p12 region. This region encompasses the *JAG1*, mutations in which are associated with Alagille syndrome. Patients with Alagille syndrome show diverse phenotypes because of variable expressivity (Goldman & Pranikoff, 2011; Guegan, Stals, Day, Turnpenny, & Ellard, 2012).

Heterozygous deletion of the *FOXA2* results in mild phenotypes and moderately decreased glucagon gene expression and α‐cell differentiation (Gao et al., 2010; Heddad Masson et al., 2014). One copy of the *FOXA2* appears to be enough for the development of a normal pancreas with all mature cell types (Ang & Rossant, 1994; Gao et al., 2008; Weinstein et al., 1994). Recently, two studies reported an association of *FOXA2* heterozygous mutations with hyperinsulinism, hypoglycemia, pituitary hormone deficiency, craniofacial, and endoderm‐derived organ abnormalities (Giri et al., 2017; Vajravelu et al., 2018).

Here, we report a 10‐year‐old boy with dysmorphic features, growth hormone (GH) deficiency, and central hypothyroidism who carries a de novo t(6;20) chromosome translocation with a heterozygous proximal 20p11.2 deletion.

## 2 CASE PRESENTATION

**Figure 1:**
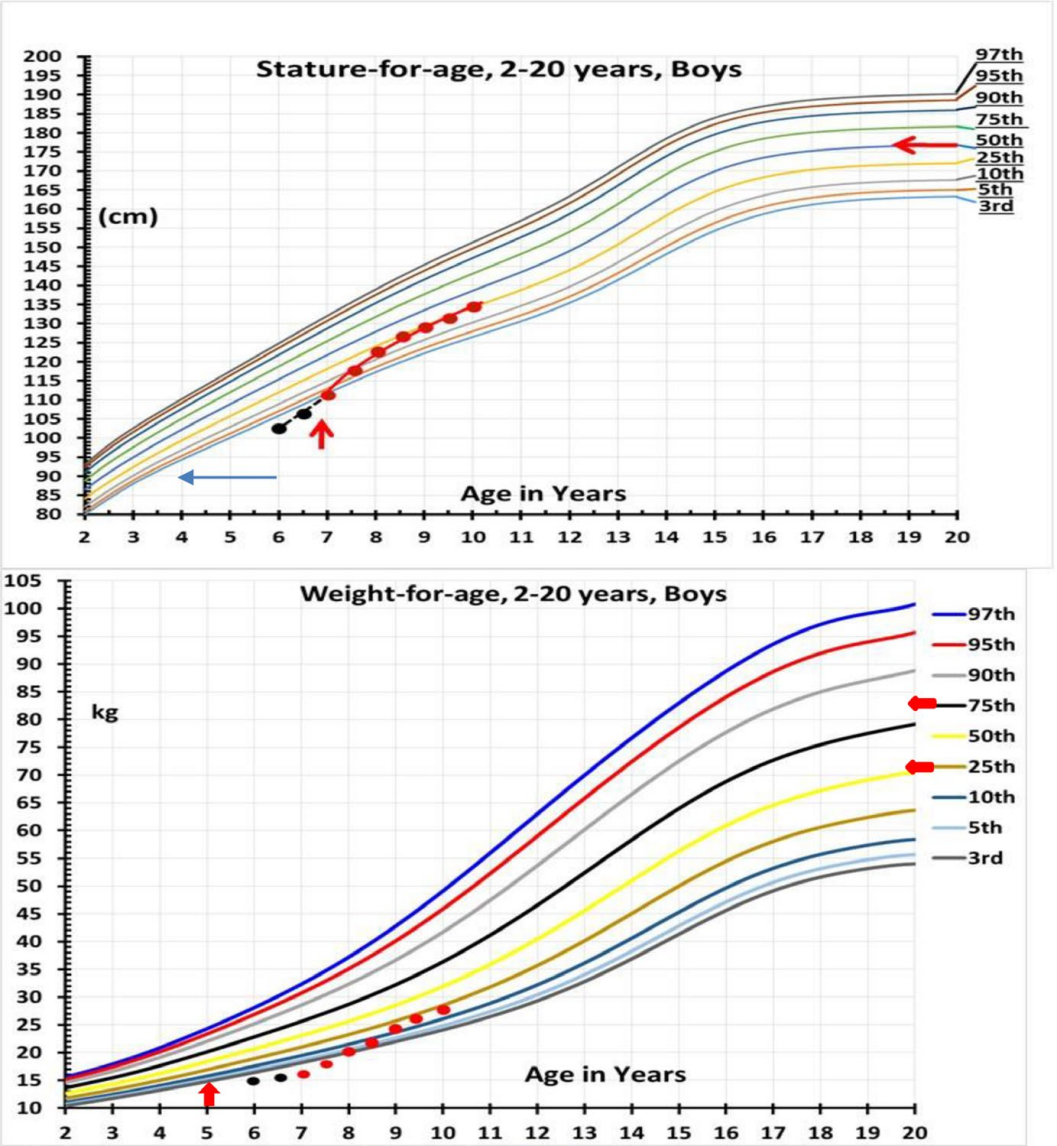
Growth chart illustration of growth abnormalities observed in the patient. Black dots, stature, and height before treatment. Red dots, stature, and height after treatment. The vertical red arrow indicates the age of commencement of growth hormone therapy. The horizontal red arrow indicates the mid‐parenteral height. Blue arrow indicates a delayed bone age of 4 years (at a chronological age of 6 years)

**Figure 2:**
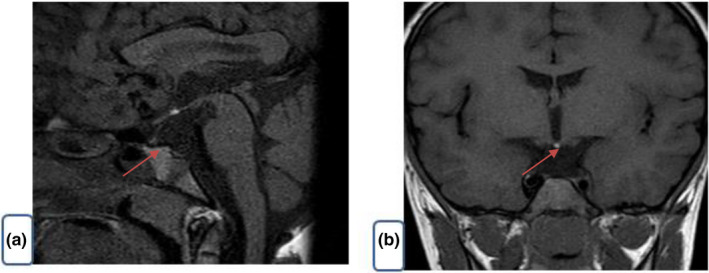
MRI scan of the brain showing abnormal imaging, (a) hypoplasia of posterior pituitary gland. (b) hypoplasia sella with ectopic posterior pituitary gland

The patient is the first child of healthy non‐consanguineous couple (mother: 21 years, father: 31 years old), with no relevant family history. The patient was born at 29 weeks of gestation by cesarean section, with low birth weight of 900g. A subsequent sister was born at term and had a normal karyotype. He presented at the age of 6 years with short stature where his height and weight at 6 years of age were two standard deviation (−2 *SD*) below the mean with a delayed bone age (Figure 1), and was noted previously to have multiple dysmorphic features. Investigations confirmed GH deficiency (Table 1), central hypothyroidism, poor weight gain, repeated episodes of abdominal pain, vomiting, and diarrhea. The patient was commenced on replacement therapy with recombinant GH (0.025 mg/kg per day with excellent response in terms of growth velocity) and levothyroxine 50 mcg daily. The serum ACTH and cortisol levels were normal. Pelvic ultrasound showed ectopic inguinal testes with the left being smaller than right. Polysplenia (three spleens) were noted on ultrasound, the largest measuring 5.5 cm in length. A barium follow through showed evidence of partial malrotation (with the third part of the duodenum incompletely crossing the midline and jejunal loops located on the right side). Contrast swallow study showed major gastroesophageal reflux, low‐lying duodenojejunal flexure with the small bowel loops seen on the right side of the abdomen suggestive of malrotation. Brain MRI scan showed that the sella turcica was shallow and underdeveloped with minor sella‐associated tissue content. These findings indicate hypoplasia of the sella and associated sella tissue (predominantly anterior pituitary) components and infundibulum (at the connection between the hypothalamus and the posterior pituitary). The physiological posterior pituitary tissue components were ectopically positioned within the region of the tuber cinereum and the lateral ventricles were slightly capacious (Figure 2). Continuous glucose patient monitoring for 7 days showed normal glucose levels, and diagnostic fast for 18 hr did not elicit any hypoglycemia. Other laboratory investigations, including serum glucagon level, kidney and liver function tests, were normal (Table 1).

**Table 1:** Biochemical investigations at time of diagnosis

**Endocrinology**			
Parameter	Result		Reference
HGH 30	0.16 μ/L	Low	7–10 μ/L
HGH 60	0.21 μ/L	Low	7–10 μ/L
HGH 90	0.18 μ/L	Low	7–10 μ/L
HGH baseline	0.17 μ/L	Low	7–10 μ/L
IGF1	46 μ/L	Low	85.2–249 μ/L
IGFBP−3	4.3 μ/L	Normal	1.6–6.5 μ/L
Glucagon	18 pg/mL	Normal	≤80 pg/Ml
TSH	0.22 mIU/L	Low	0.3–5 mIU/L
FT4	9.3 pmol/L	Low	11.58–18 pmol/L
**Immunology and autoimmune disease**			
IgG	1,390 mg/dl	High	633–1280 mg/dl
IgA	251 mg/dl	High	33–202 mg/dl
Anti‐trasglutaminase IgA Ab	Negative		–
Anti‐trasglutaminase IgGAb	Negative		–

## 3 MATERIALS AND METHODS

### 3.1 Molecular karyotyping

DNA was extracted from a peripheral blood sample of the patient using QIAsymphony DSP DNA Kits according to the manufacturer's instructions. Genome‐wide copy number analysis was performed using an Affymetrix CytoScan 750K Array according to the manufacturer's instructions. Regions of copy number change were determined using the Affymetrix Chromosome Analysis Suite software (ChAS) v.3.2 and interpreted with the aid of the UCSC genome browser (http://genome.ucsc.edu/; Human February 2009 GRCh37/hg19 assembly).

In the case of parental samples, 1 μg of DNA and opposite‐sex commercially available control DNA were labeled with Cy3‐ or Cy5‐deoxycytidine triphosphate, respectively, using Exo‐Klenow (OGT Cytosure Labeling Kit, catalogue no. 020020), and hybridized to a CytoSure ISCA 8 × 60 k array at 65°C for 18 hr. The array was washed and scanned with the Agilent High‐Resolution Microarray Scanner Model G2505B at 3‐µm resolution. Data analysis was performed using Feature Extraction version software from Agilent technologies and CytoSure Interpret Software (OGT).

### 3.2 Conventional cytogenetics

A cytogenetic study was performed to characterize molecular karyotype findings. Chromosome analysis was performed according to standard procedures on metaphase cells obtained from short‐term culture of the peripheral blood sample. At least 20 metaphases were observed and analyzed by GTG banding with over 550 band resolution observed using ikaros software from metasystem. The karyotype was designated according to the International System for human Cytogenetic Nomenclature (ISCN).

### 3.3 DNA sequencing

In the case of Sanger‐based sequencing, the mRNA sequence of the *FOXA2* was identified using the UCSC genome browser (http://genome.ucsc.edu). This website provides a direct link to ExonPrimer for the design of primers flanking coding exons. All primers were checked for single‐nucleotide polymorphisms (SNPs) using the software tool available from the National Genetic Reference Laboratory, Manchester (https://ngrl.manchester.ac.uk/SNPCheckV3/snpcheck.htm). The primers were tailed with M13 sequences and were synthesized by Invitrogen Ltd.

PCR was performed using 1U Faststart Taq DNA polymerase (Invitrogen Ltd), 50 ng genomic DNA, 2‐mM MgCl2, 0.8‐μM forward and reverse primers, with the following cycle conditions: 95°C for 4 min, 35 cycles of 94°C for 45s, 60°C for 30 s, 72°C for 30 s, and a final extension at 72°C for 10min. All amplicons amplified efficiently under these conditions. Five microliter of each PCR was cleaned with ExoSAP‐IT (Affymetrix) prior to bidirectional DNA sequencing using M13 forward and reverse primers and Big‐Dye Terminator v3.0 (Applied Biosystems Ltd). Twenty microliter of sequenced product was purified using Clean‐Seq (Agencourt). Fifteen microliter of purified product was then subjected to capillary electrophoresis using an Applied Biosystems model 3500 Genetic Analyzer. Sequencing analysis software v6.0 and SeqScape software v3.0 were used for Sanger sequencing analysis (Applied Biosystems, Cat No.: 4474978).

In the case of whole genome sequencing (WGS), DNA was extracted from blood from the patient and his parents. As a first step, the extracted DNA was subjected to WGS using an Illumina HiSeqX platform and a 150‐base paired‐end single‐index‐read format. This method yielded two FASTQ files that contained the nucleotide sequence reads and quality scores for each sample. Genome Analysis Toolkit (GATK) version 3.6 was used to further process the FASTQ files in order to identify variants in the patient and parents. FastQC (version 0.11.2) software was run on the raw data, together with combinations of SAM Tools (version 1.7). Burrows–Wheeler Aligner 0.7.8 (BWA‐MEM) was used to map the sequence reads to the NCBI human reference genome GRGh37/hg19. Variants were then called using the GATK tools which produce a variant call format (VCF) file that gives information on SNPs, indels (insertion or deletions), and other structural variants in the samples that were processed.

## 4 RESULTS

### 4.1 Molecular karyotype

Molecular karyotype analysis of the proband showed a male profile with an interstitial deletion of the short arm of chromosome 20: arr[hg19] 20p11.22p11.21(21881142–22850635)X1. The deletion of ~969 kb contains only one OMIM gene: *FOXA2*. Each parent had a normal molecular karyotype profile suggesting the deletion observed in our patient was de novo.

### 4.2 Conventional cytogenetics

**Figure 3:**
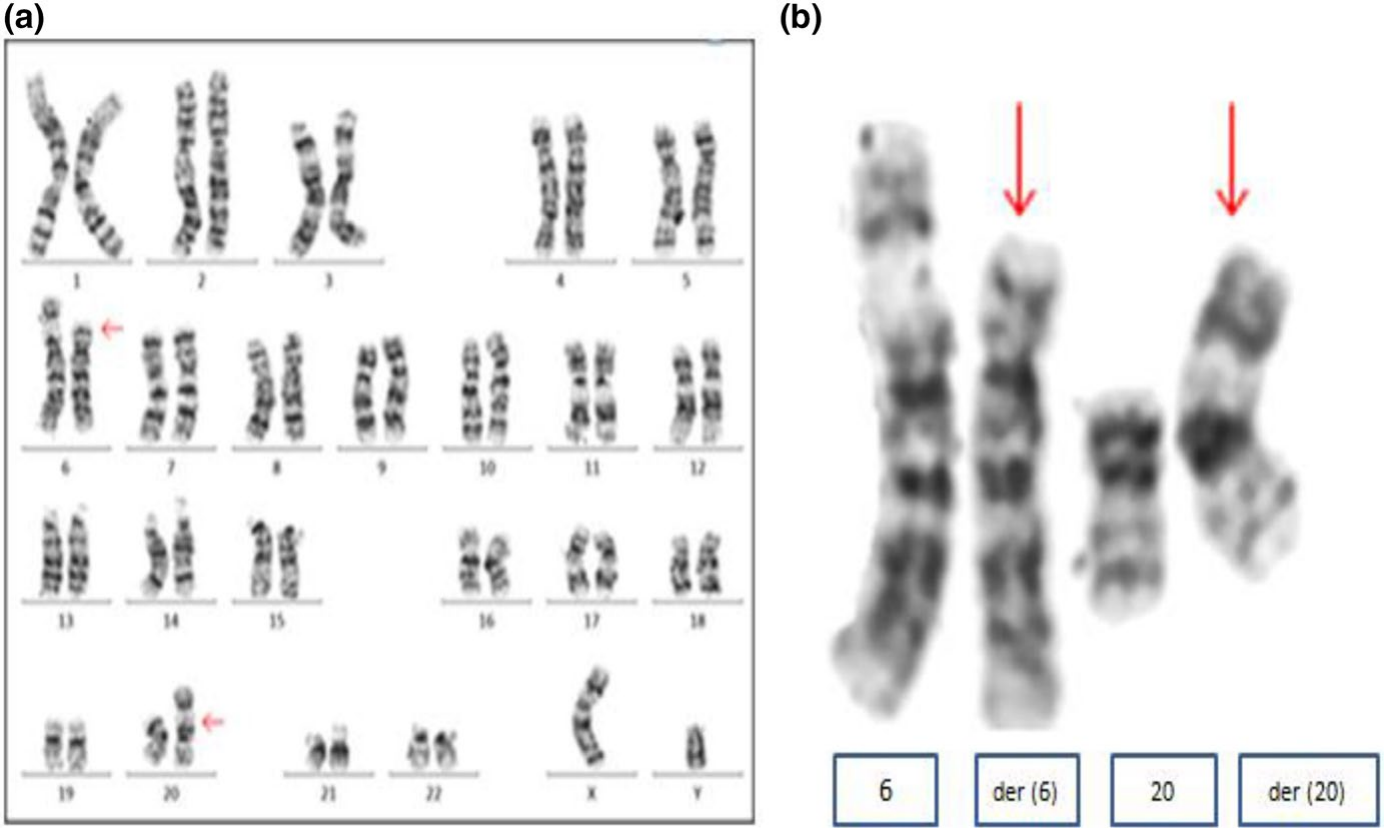
Conventional karyotyping revealing translocation between chromosome 6 and 20. a) A male karyotyping with t(6;20)(p11.2;p11.2). b) Partial karyotype showing t(6;20)(p11.2;p11.2)

G‐banding chromosome analysis of 20 cells from a peripheral blood sample of the proband revealed a male chromosome complement with a reciprocal translocation between the short arm of chromosome 6 and 20, karyotype: [46 XY, t (6; 20) (p11;p11)] (Figure 3). Each parent had a normal karyotype.

### 4.3 DNA Sequencing

Sanger‐based sequence analysis showed no variants in the coding regions of the *FOXA2* of our patient compared to controls and the human genome reference.

We undertook WGS analysis of our patient, together with parental samples, with a focus on those genes implicated in combined pituitary hormone deficiency, GH deficiency, and hypothyroidism. The gene list was constructed by searching a number of databases: NCBI dbSNP, ClinVar, 1000 genome, Human Genome Variation Society (HGVS), and Ensembl. No pathogenic or likely pathogenic single nucleotide variants were detected in our patient, or his parents. A screen for copy number variants (CNVs) identified a large deletion encompassing the *FOXA2* with a depth of coverage of 15X in our patient compared to an average of 30X, suggesting a heterozygous deletion of the *FOXA2*. No obvious CNVs were detected in chr6p11.

## 5 DISCUSSION

Most of the previously reported deletions of chromosome 20 short arm, involving the 20p11.2 region, are associated with Alagille syndrome. This deleted region includes the *JAG1*. Alagille syndrome is inherited in an autosomal dominant manner and affects various organs with variable severity due to variable expressivity (Anad et al., 1990; Elmslie et al., 1995).

Our patient's genotype at the whole genome level was characterized as 46, XY,t(6;20)(p11.2;p11.2) and arr[hg19] 20p11.22p11.21(21881142–22850635)X1. The deletion only encompasses the *FOXA2*. The main phenotypes associated with deletions in this region are panhypopituitarism and GH deficiency. Five of the eight reported cases overlapping with our case presented with panhypopituitarism (Dayem‐Quere et al., 2013; Kale et al., 2017; Tsai et al., 2015; Williams, Wetherbee, Rosenfeld, & Hersh, 2011). Our patient had severe GH deficiency and central hypothyroidism that required treatment (levothyroxine and GH therapy). It is not surprising that patients who were reported earlier (Garcia‐Heras et al., 2005; Kamath et al., 2009; Tsai et al., 2015) presented with GH deficiency and shared a deleted region partially overlapping with our case (Table 2).

**Table 2:** Genomic coordinates in our patient and previously reported case of chromosome 20p deletion (GRCH37, hg19)

Authors	hg19 coordinates	
This patient	21881142	22850635
Dayem‐Quere et al., 2013	19810034	24031344
Garcia‐Heras et al., 2005	No MKAR data, only G‐banding: 20p11.1−20p12	
Kale et al., 2017	(build 36) hg18: 15841592 15893592	Build 36 (hg18): 24189610 24241610
Kamath et al., 2009	13618382 (build 35/hg17: 13566382) 14352641 (build 35/hg17: 14300641)	22303261 (build 35/hg17: 22251261) 26309255 (build 35/hg17: 26257255)
Laufer‐Cahana et al., 2002	No MKAR data, only G‐banding	
Michaelis et al., 1997	D20S80 not deleted: D20S104 deleted: 16187053 16187053	PCSK2 deleted: 17465222 D20S40 not deleted 17465222
Tsai et al., 2015	22496147	22773103
Williams et al., 2011	(build 36) hg18: 19149641 19201641	(build36) hg18: 24569358 24621358

**Figure 4:**
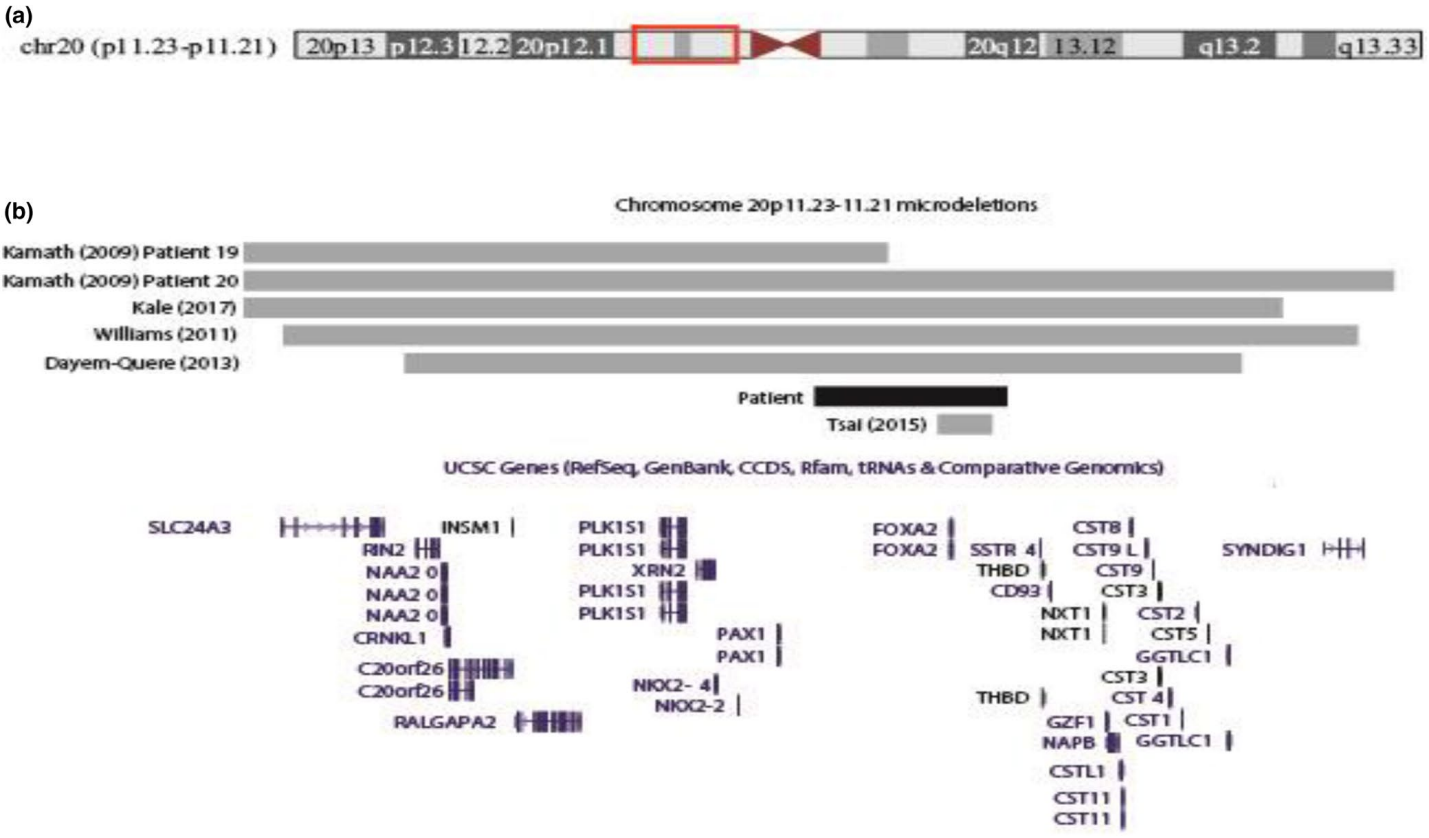
Location and extent of microdeletions reported in chr20p11.23–11.21. (a) Shows an ideogram of chromosome 20. (b) Shows the location and extent of the deletions detected in the patient described here, and other cases reported in the literature, as well as Refseq genes that lie within the shown region. In the case of the deletions shown for patient 19 reported by Kamath et al. (2009), and the deletion shown for the patient reported by Kale et al. (2017), these extend further distally, while the deletion shown for patient 20 reported by Kamath et al. (2009) extends both proximally as well as distally. These graphics were taken from the UCSC genome browser (http://genome.ucsc.edu/)

Recently, de novo heterozygous *FOXA2* mutations have been reported in patients with the clinical phenotype of congenital hypopituitarism, hyperinsulinism, hypoglycemia, and endometrial‐derived organ abnormalities (Giri et al., 2017; Tsai et al., 2015; Vajravelu et al., 2018). Our patient shares most of these phenotypes except that our patient does not exhibit hyperinsulinemic hypoglycemia. A recent report identified a de novo heterozygous mutation in the *FOXA2*, c.664T> G (p.Cys222Gly), in a patient with intestinal malrotation, anal atresia, and pituitary hormone deficiency (Boda et al., 2018; Figure 4).

To understand if our patient had any hypoglycemia, we undertook continuous glucose monitoring in our patient for 7 days and there was no hypoglycemia. The patient also fasted for 18 hr with no hypoglycemia and appropriate increases in serum fatty acids and ketone bodies. Similarly, the serum glucagon concentration of our patient was within the normal reference range. These results of the glucose and glucagon levels suggest that one copy of *FOXA2* may be sufficient to maintain normal blood glucose, insulin, and glucagon levels. However, it is unclear if the intellectual, developmental, and metabolic status of the child may change with age.

## 6 CONCLUSIONS

**Figure 5:**
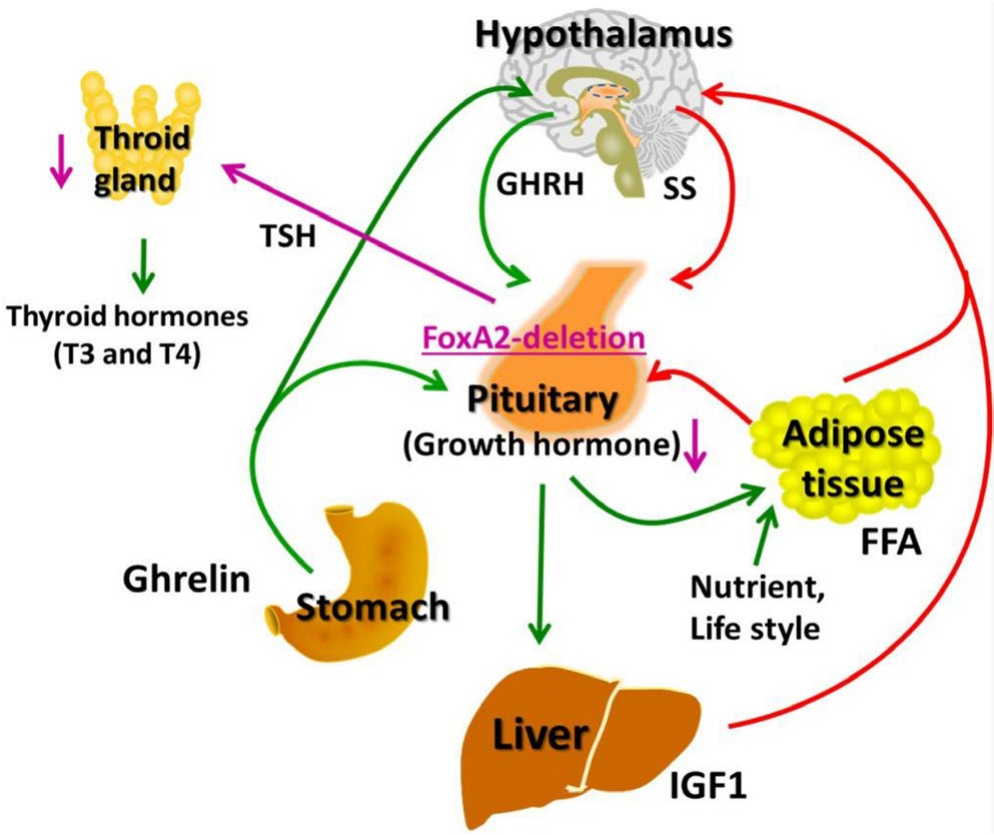
Factors that stimulate and suppress GH secretion under physiological conditions. Green indicates stimulation and red indicates inhibition. In the patient, *FOXA2* deletion lead to growth hormone deficiency, hypothyroidism, and reduced weight gain. TSH: Thyroid‐stimulating hormone; FFA: Free fatty acid; IGF1: Insulin‐like growth factor‐1; SS: Somatostatin; GHRH: Growth hormone–releasing hormone

Our patient is the first reported case of an unbalanced t(6;20) translocation carrying a small chr20p deletion involving only the *FOXA2*, and exhibiting features related to GH deficiency and hypothyroidism (Figure 5). Our patient's clinical findings, together with previously reported cases, suggest that haploinsufficiency of *FOXA2* has implications in panhypopituitarism and GH deficiency. Panhypopituitarism may be the most common feature represented in heterozygous 20p deletions caused by the shallow and underdeveloped sella and pituitary hormonal deficiency. Patients with heterozygous *FOXA2* mutations appear to exhibit hyperinsulinemia and hypoglycemia. Our patient has not manifested any changes in blood glucose, glucagon, or insulin levels, but he may develop later‐onset diabetes. It is possible that the complete loss of *FOXA2* affects the pancreas and glucose levels, whereas haploinsufficiency of *FOXA2* affects mainly the pituitary gland.

Studying the signaling pathways affected by the heterozygous loss of *FOXA2,* as well as identifying the upstream and downstream protein partners of *FOXA2*, may allow an understanding of how *FOXA2* haploinsufficiency leads to GH deficiency and hypopituitarism.

## ETHICS STATEMENT

Written informed consent was obtained from the parents of the patient for the participation in the study and the publication of this case report and accompanying images. A copy of the written consents from both parents is available for review by the editor of this journal.

## CONFLICT OF INTEREST

The authors declare that the research was conducted in the absence of any commercial or financial relationships that could be construed as a potential conflict of interest.
